# Clinical and Metabolic Factors as Predictors of Thirty-Day Readmission in Patients With Acute Decompensated Heart Failure With Reduced Ejection Fraction and Type 2 Diabetes Mellitus

**DOI:** 10.14740/cr2206

**Published:** 2026-08-31

**Authors:** Rarsari Soerarso, Dian Yaniarti Hasanah, Emir Yonas, Azlan Sain, Sunu Budhi Raharjo, Bambang Budi Siswanto, Maarten J.M. Cramer, Pim van der Harst, Marish I.F.J. Oerlemans

**Affiliations:** a Department of Cardiology and Vascular Medicine, Faculty of Medicine, Universitas Indonesia, National Cardiovascular Center Harapan Kita, Jakarta, Indonesia; b Faculty of Medicine, Universitas YARSI, Jakarta, Indonesia; c Department of Cardiology, University Medical Center Utrecht, University of Utrecht, Netherlands; d Division of Heart and Lungs, University Medical Center Utrecht, University of Utrecht, Netherlands

**Keywords:** Heart failure, Ejection fraction, Type-2 diabetes mellitus, 30-day readmission

## Abstract

**Background:**

Patients with heart failure with reduced ejection fraction (HFrEF) had higher readmission rates than those with normal ejection fractions, and readmission rates were highest in the first 30-days post-admission. About 30% of patients with decompensated heart failure also have type 2 diabetes mellitus (T2DM). The aim of the study was to determine the clinical and metabolic predictors of 30-day readmission in patients with acute decompensated heart failure (ADHF) with reduced ejection fraction and T2DM.

**Methods:**

The study was conducted in a retrospective-cohort design, and data were taken from medical records based on the admissions of patients. The clinical outcomes were divided into readmission and non-readmission groups. The clinical outcome assessed was the incidence of readmission due to worsening of the condition of heart failure at 30 days after the last admission at the National Cardiovascular Center Harapan Kita (NCCHK), based on clinical encounters/readmission of patients. Logistic regression multivariate analysis was performed to determine significant predictors for 30-day readmission. Bootstrapping was performed to ensure robustness of the multivariate logistic regression analysis.

**Results:**

There were 747 clinical encounters, consisting of 179 readmission events, and 568 were non-readmission events (readmission rate 24%). Atrial fibrillation (AF) was found in 15.7% of clinical encounters. The median ejection fraction (EF) was 23% during admission. median fasting blood sugar and postprandial blood sugar of study subjects were 107(34–324) and 145 (51–409) mg/dL. Multivariate logistic regression analysis showed that the factors associated at 30-days readmission were: AF rhythm (odds ratio (OR) = 2.616; 95% confidence interval (CI), 1.604–4,267; P = 0.000), heart rate at discharge (area under the receiver operating characteristic (ROC) curve (AUC) 0.650 (95% CI, 0.545–0.756) P = 0.007) (OR = 1.022; 95% CI, 1.005–1.039; P = 0.010). Postprandial blood glucose level ≤ 140 mg/dL was a protective predictor for 30-day readmission (OR = 0.528; 95% CI, 0.348–0.802; P = 0.003).

**Conclusions:**

This study showed that clinical factors such as AF and increased heart rate at discharge were associated with rehospitalization risk in 30 days in patients with HFrEF and T2DM, while a metabolic factor of postprandial blood sugar ≤ 140 mg/dL was observed to have protective effects against rehospitalization in 30 days in patients with HFrEF and T2DM. ROC analysis showed that reducing discharge heart rate to below 78 beats per minute (bpm) might be beneficial in diabetic patients with HFrEF and AF.

## Introduction

Acute decompensated heart failure (ADHF) is defined as a sudden worsening of signs and symptoms of chronic heart failure (HF) [1]. Patients with HF, apart from having high mortality, are also affected by high rehospitalization rates. This is especially true in patients with heart failure with reduced ejection fraction (HFrEF). The Atherosclerosis Risk in Communities Study (ARIC) showed higher readmission rates in 30 days after initial discharge in HF patients with reduced ejection fraction (EF) compared with preserved EF (115 vs 88 readmissions per 100 person-years) [2]. In Indonesia, 29% of patients with HF are readmitted within 30 days of discharge. This phenomenon has a significant economic impact, with HF admission cases contributing to the largest cost of hospitalization compared to other cardiovascular disorders, highlighting the need to investigate the clinical characteristics and readmission profiles in ADHF with diabetes mellitus (DM) patients to better depict ADHF patient profile and clinical course in the Southeast Asian population [3, 4].

Patients with HF are commonly found to also suffer from type 2 diabetes mellitus (T2DM). Almost 70% of patients with HF suffer from either prediabetes or T2DM. At least a third of admitted HF patients suffer from type 2 diabetes [5]. Patients with type 2 diabetes are found to be at a twofold risk of developing HF compared to patients without diabetes. HF patients with type 2 diabetes also showed higher admission and readmission rates compared to patients without diabetes. In patients with HF, diabetes and other conditions contributed to the progressivity, complexity, and severity of HF.

A large study of HF patients involving 1,501,811 patients with HFrEF, of whom 36.87% had T2DM, showed a fivefold increase in admission risk in patients who are diabetic compared to nondiabetic patients [6].

The factors associated with increased mortality and rehospitalization risk in HFrEF patients with T2DM remain to be investigated, especially in the first 30 days following initial discharge from the hospital.

Several risk assessment scoring systems, including the LACE index (length of stay, acuity of admission, comorbidity, and previous presentations to emergency), the RAHF (readmission after heart failure) score, and the DERRI (diabetes early readmission risk indicator), have been studied. However, these scoring systems only represent risk assessment on Western populations and often do not resemble phenomenon seen in non-Western countries [5, 7]. Currently, there are no studies that specifically investigated the factors that are associated with rehospitalization and death in specific HFrEF patients who also suffer from T2DM. The objective of this study is to investigate clinical and metabolic factors that are associated with readmission within 30 days in patients with HFrEF and T2DM.

## Materials and Methods

### Study design

This is a retrospective cohort study of acutely decompensated HF patients with decreased EF and T2DM who were hospitalized at the National Cardiovascular Center Harapan Kita, Jakarta, Indonesia, between January 1, 2016, and February 1, 2021.

The inclusion criteria for this study were patients with ADHF and decreased EF (EF ≤ 40%) who had been diagnosed with T2DM and who were admitted between January 1, 2016, and February 1, 2021.

The exclusion criteria were as follows: (1) acute coronary syndrome, including ST-segment elevation myocardial infarction (STEMI), non-ST-segment elevation myocardial infarction (NSTEMI), and unstable angina pectoris (UAP); (2) ADHF with cardiogenic shock clinical profile, including acute lung edema and isolated right HF; (3) primary valvular heart disease; (4) congenital heart disease; (5) a history of valvular surgery; (6) hypertrophic obstructive cardiomyopathy/restrictive cardiomyopathy; (7) death during hospitalization; (8) referral to another hospital; (9) discharge against medical advice; and (10) inability to be contacted by telephone.

Data were collected retrospectively from the medical records of the National Cardiovascular Center, Harapan Kita.

Independent variables of this study consisted of both clinical and metabolic factors: (1) age; (2) gender; (3) body mass index (BMI); (4) blood pressure; (5) heart rate (HR); (6) atrial fibrillation (AF)/ventricular tachycardia; (7) pleural effusion; (8) infection during hospitalization; (9) hemoglobin level; (10) use of intensive diuretic therapy; (11) use of other diuretics; (12) use of angiotensin-converting enzyme inhibitor (ACE-I), angiotensin receptor blocker (ARB), and angiotensin receptor neprilysin inhibitor (ARNI) regiments; (13) use of mineralocorticoid receptor antagonist (MRA) regiment; (14) use of beta-blocker regiment; (15) use of hypoglycemic agents; (16) EF; (17) kidney function; (18) blood sugar levels; and (19) serum electrolyte levels.

The dependent variable and primary outcome of interest of this study are readmission in 30 days secondary to worsening HF.

### Data analysis

The primary unit of analysis and outcome of interest is readmission based on clinical encounter, which reflects the dynamic nature of clinical progression of HF with DM at each admission. Data analysis begins with an assessment of data distribution. Assessment of the normality of distribution in numerical data was conducted using the Kolmogorov–Smirnov test. Categorical data were presented using percentages, and numerical data were presented with mean ± standard deviation (SD) for normally distributed data and median with the minimum–maximum value for non-normally distributed data. Several variables were categorized using our operational definition. Bivariate analyses were done using Chi-square test for categorical data. Mann–Whitney U test was used for numerical data with non-normal distribution, and the independent sample *t*-test for numerical data with normal distribution. Relative risk calculation with 95% confidence interval (CI) was performed for categorical data. Variables with missing data ≥ 10% were excluded from the analysis. Several variables were dichotomized for analysis. Fasting blood glucose was dichotomized into ≤ 100 and ≥ 100 mg/dL, and postprandial glucose level into ≤ 140 and ≥ 140 mg/dL, to align with established recommendations of the American Diabetes Association and Indonesian Society of Endocrinologists, therefore providing highly actionable clinical targets for ward physicians. All variables with a P value ≤ 0.25 in baseline analysis are included in multivariate analysis (logistic regression). To ensure the reproducibility of the internal validation, bootstrapping was performed with 1,000 resamples utilizing a fixed random seed (Mersenne Twister seed: 20000) to calculate bias-corrected and accelerated (BCa) 95% CIs. A P value lower than 0.05 was used as a threshold of statistical significance in this study. All analyses were conducted using SPSS version 26 (IBM Statistics, USA).

### Ethical clearance

This study was approved by the National Cardiovascular Center Harapan Kita Research Ethics Committee (approval letter number: LB.02.01/VII/519/KEP 013/2021). The study was conducted in accordance with the ethical principles of the Declaration of Helsinki.

## Results

Initially, 1,079 clinical encounters of patients with HFrEF and T2DM were identified. After applying exclusion criteria, a total of 747 clinical encounters remained for final analysis. This consisted of 179 readmission events and 568 non-readmission events. The majority of these encounters involved males (74.8%) with a median age of 58 years. The median length of stay (LOS) across all encounters was 6 (1–76) days. The incidence of infection during admission was 65.6%. Only a few patients (44.5%) reported good adherence to medication regimens. Pleural effusion was present in 13.8% of admissions, and 19.7% of admissions were complicated by chronic kidney disease (CKD) at presentation. In this study, 92.4% of admissions were under an ACE-I/ARB/ARNI regimen, 95.2% under a loop diuretic, 68.3% under an MRA, and 82.3% clinical encounters under a beta-blocker regimen. Other types of diuretics, such as thiazides, were only used by 9.31% of patients on admission. Most of the study subjects received oral hypoglycemic agents (OHO), including insulin on discharge (90.4%). Table 1 shows baseline characteristics of study subjects.

**Table 1:** Baseline Characteristics of Study Subjects

Variables	Missing data (%)	Total sample (n = 747)
Gender	0	
Male, n (%)		559 (74.8%)
Female, n (%)		188 (25.2%)
Age (years), median (Min–Max)		58 (31–88)
No. of hospitalizations	0	
1–5, n (%)		530 (71%)
6–10, n (%)		135 (18.1)
11–15, n (%)		46 (6.2%)
> 15, n (%)		36 (4.8%)
LOS (days), median (Min–Max)	0	6 (1–76)
Infection during admission	0	
Yes, n (%)		490 (65.6%)
No, n (%)		257 (34.4%)
Medication adherence	358 (47.9%)	
Good, n (%)		173 (44.5%)
Poor, n (%)		216 (55.5%)
Pleural effusion	0	
Yes, n (%)		103 (13.8%)
No, n (%)		644 (86.2%)
Chronic kidney disease	0	
Yes, n (%)		147 (19.7%)
No, n (%)		600 (80.3%)
ACE-I/ARB/ARNI on discharge	0	
Yes, n (%)		690 (92.4%)
No, n (%)		57 (7.6%)
Intensive diuretic on discharge	0	
Yes, n (%)		711 (95.2%)
No, n (%)		36 (4.8%)
MRA on discharge	0	
Yes, n (%)		510 (68.3%)
No, n (%)		237 (31.7%)
Other diuretic at discharge	0	
Yes, n (%)		68 (9.1%)
No, n (%)		679 (90.9%)
Beta-blocker at discharge	0	
Yes, n (%)		615 (82.3%)
No, n (%)		132 (17.7%)
Oral hypoglycemic agents at discharge	0	
Yes, n (%)		675 (90.4%)
No, n (%)		72 (9.6%)
ECG during admission	0	
SR, n (%)		597 (79.9%)
AF, n (%)		117 (15.7%)
VT, n (%)		9 (1.2%)
Other, n (%)		24 (3.2%)
Ejection fraction (%), median (Min–Max)	14 (1.9%)	23(12–39)
Systolic blood pressure (mm Hg), median (Min–Max)	0	101 (69–187)
Diastolic blood pressure (mm Hg), median (Min–Max)	0	65 (29–115)
Heart rate (bpm), median (Min–Max)	0	77 (50–115)
Body weight (kg), median (Min–Max)	47 (6.3%)	65.5 (36.5–142.85)
Body mass index (kg/m^2^), median (Min–Max)	44 (5.9%)	24.08 (15.43–48.29)
Fasting blood glucose (mg/dL), median (Min–Max)	14 (1.9%)	107 (34–324)
Postprandial blood glucose (mg/dL), median (Min–Max)	13 (1.7%)	145 (51–409)
Kidney function panel		
Urea (mg/dL), median (Min–Max)	8 (1.1%)	65.9 (15.3–231.30)
BUN (mg/dL), median (Min–Max)	11 (1.5%)	31 (7–109)
Creatinine (mg/dL), median (Min–Max)	11 (1.5%)	1.47 (0.45–9.66)
GFR (mL/min/1.73 m^2^), median (Min–Max)	14 (1.9%)	46 (4–133)
Hemoglobin (g/dL), mean ± SD	16 (2.1%)	13.05 ± 2.12
Sodium (mmol/L), median (Min–Max)	3 (0.4%)	137 (113–147)
Potassium (mmol/L), median (Min–Max)	3 (0.4%)	4 (2.7–6.0)
Chloride (mmol/L), median (Min–Max)	3 (0.4%)	95 (72–111)
Calcium (mmol/L), median (Min–Max)	174 (23.3%)	2.22 (1.74–2.66)
Magnesium (mmol/L), median (Min–Max)	157 (21.0%)	2.0 (1.2–3.1)

Numerical data with abnormal distribution were presented with median and Min–Max, while numerical data with normal distribution were presented with mean and SD. Categorical data were presented in percentages. SD: standard deviation; bpm: beat per minute; Min: minimum; Max: maximum; ACE-I: angiotensin-converting enzyme inhibitor; ARB: angiotensin receptor blocker; ARNI: angiotensin receptor neprilysin inhibitor; LOS: length of stay; BUN: blood urea nitrogen; ECG: electrocardiogram; GFR: glomerular filtration rate; SR: sinus rhythm; VT: ventricular tachycardia; AF: atrial fibrillation; MRA: mineralocorticoid receptor antagonist.

AF was found in 15.7% of clinical encounters during admission. The median EF of study subjects in this study was 23% during admission. Regarding DM, the median fasting blood sugar and postprandial blood sugar of study subjects were 107 (34–324) and 145 (51–409) mg/dL. The median glomerular filtration rate (GFR) of study subjects was 46 mL/min/1.73 m^2^. In this study, there were three variables with missing data ≥ 10%, namely medication adherence, calcium, and magnesium levels (Table 1).

On bivariate analysis, statistically significant results in categorical data were seen on AF (24% vs 13%, P = 0.001), ACE-I/ARB/ARNI on discharge (88.3% vs 93.7%, P = 0.018), fasting blood glucose ≥ 100 mg/dL (70.0% vs 59.5%, P = 0.013), and postprandial glucose ≥ 140 mg/dL on discharge (68.4% vs 52%, P ≤ 0.001). Statistically significant results in numerical data were seen on systolic blood pressure (SBP) 106 (69–164) vs 101 (69–187) mm Hg, HR 81 (50–114) vs 72 (51–105) beats per minute (bpm), urea level 72 (19.7–231.3) vs 62.2 (15.3–207.5) mg/dL, and blood urea nitrogen (BUN) level 33.5 (9–109) vs 29 (7–97) mg/dL.

Based on the results of bivariate analysis, variables that are eligible to be included in multivariate analysis are gender (P = 0.170), infection on admission (P = 0.239), AF on admission (P = 0.001), use of ACE-I/ARB/ARNI on discharge (P = 0.0018), beta-blocker on discharge (P = 0.098), fasting blood glucose ≥ 100 mg/dL (P = 0.013), postprandial glucose ≥ 140 mg/dL (P ≤ 0.001), age (P = 0.054), SBP (P = 0.005), HR (P ≤ 0.001), BUN (P ≤ 0.001), and EF (P = 0.171). Tables 2 and 3 show the results of bivariate analysis of categorical and numerical variables.

**Table 2:** Bivariate Analysis of Independent Categorical Variables to the Incidence of Heart Failure Readmission in 30 Days

Variables	Readmission/yes, n (%)	Readmission/no, n (%)	P value	RR	95% CI	
					Min	Max
Gender						
Male	127 (70.9)	432 (76.1)	0.170	1.217	0.923	1.606
Female	52 (29.1)	136 (23.9)				
Infection during admission						
Yes	47 (26.3)	125 (22.0)	0.239	1.191	0.894	1.585
No	132 (73.7)	443 (78.0)				
Pleural effusion						
Yes	21 (11.7)	82 (14.4)	0.360	0.830	0.554	1.245
No	158 (88.3)	486 (85.6)				
CKD						
Yes	36 (20.1)	111 (19.5)	0.867	1.029	0.895	1.097
No	143 (79.9)	457 (80.5)				
Adherence						
Good	51 (53.1)	122 (41.6)	0.049	0.706	0.499	1.000
Poor	45 (46.9)	171 (58.4)				
ECG during admission						
SR	129 (72.1)	468 (82.4)	0.005		—	—
AF	43 (24.0)	74 (13.0)	0.001	2.108	1.380	3.219
VT	3 (1.7)	6 (1.1)	0.404	1.814	0.448	7.353
Other	4 (2.2)	20 (3.5)	0.564	0.726	0.244	2.160
ACE-I/ARB/ARNI on discharge						
Yes	158 (88.3)	532 (93.7)	0.018	0.622	0.431	0.897
No	21 (11.7)	36 (6.3)				
Intensive diuretic on discharge						
Yes	170 (95.0)	541 (95.3)	0.881	0.956	0.535	1.710
No	9 (5.0)	27 (4.7)				
Other diuretics on discharge						
Yes	14 (7.8)	54 (9.5)	0.494	0.847	0.521	1.376
No	165 (92.2)	514 (90.5)				
Beta-blocker on discharge						
Yes	140 (78.2)	475 (83.6)	0.098	0.770	0.570	1.041
No	39 (21.8)	93 (16.4)				
MRA on discharge						
Yes	127 (71.0)	383 (67.4)	0.378	1.135	0.855	1.507
No	52 (29.0)	185 (32.6)				
OHO/insulin on discharge						
Yes	163 (91.1)	512 (90.1)	0.716	1.087	0.691	1.708
No	16 (8.9)	56 (9.9)				
Fasting blood glucose (mg/dL)						
≥ 100	121 (70.0)	333 (59.5)	0.013	1.430	1.071	1.909
< 100	52 (30.0)	227 (40.5)				
Postprandial glucose (mg/dL)						
≥ 140	119 (68.4)	291 (52.0)	0.000	1.710	1.287	2.272
< 140	55 (31.6)	269 (48.0)				

ACE-I: angiotensin-converting enzyme inhibitor; AF: atrial fibrillation; ARB: angiotensin receptor blocker; ARNI: angiotensin receptor neprilysin inhibitor; CKD: chronic kidney disease; ECG: electrocardiogram; SR: sinus rhythm; VT: ventricular tachycardia; MRA: mineralocorticoid receptor antagonist; RR: risk ratio; CI: confidence interval; Min: minimum; Max: maximum; OHO: oral hypoglycemic agents

**Table 3:** Bivariate Analysis of Independent Numerical Variables on Incidence of Heart Failure (HF) Readmission in 30 Days

Variable	HF readmission (n = 179)	No readmission (n = 568)	P value
Age (years), median (Min–Max)	59 (40–77)	58 (31–88)	0.054
SBP (mm Hg), median (Min–Max)	106 (69–164)	101 (69–187)	0.005
DBP (mm Hg), median (Min–Max)	66 (33–109)	65 (29–115)	0.173
Heart rate (bpm), median (Min–Max)	81 (50–114)	72 (51–105)	0.000
Body weight (kg), median (Min–Max)	65 (38.45–128)	65.8 (36.5–142.85)	0.776
BMI (kg/m^2^), median (Min–Max)	23.94 (15.82–45.90)	24.16 (15.43–48.29)	0.476
Fasting blood glucose (mg/dL), median (Min–Max)	113 (40–321)	104 (34–324)	0.015
Post–prandial glucose (mg/dL), median (Min–Max)	158.5 (80–372)	142.5 (51–409)	0.002
Urea (mg/dL), median (Min–Max)	72 (19.7–231.3)	62.2 (15.3–207.5)	0.001
BUN (mg/dL), median (Min–Max)	33.5 (9–109)	29 (7–97)	0.001
Creatinine (mg/dL), median (Min–Max)	1.49 (0.52–8.76)	1.46 (0.45–9.66)	0.760
GFR (mL/min/1.73 m^2^), median (Min–Max)	46 (5–112)	46 (4–133)	0.698
Sodium (mmol/L), median (Min–Max)	137 (121–144)	137 (113–147)	0.578
Potassium (mmol/L), median (Min–Max)	4.0 (2.7–5.5)	4.0 (2.8–6)	0.387
Chloride (mmol/L), median (Min–Max)	95 (78–109)	95 (72–111)	0.260
Calcium (mmol/L), median (Min–Max)	2.22 (1.90–2.54)	2.22 (1.74–2.66)	0.687
Magnesium (mmol/L), median (Min–Max)	2.0 (1.30–2.80)	2.0 (1.20–3.10)	0.144
Ejection fraction (%), median (Min–Max)	22.15 (12.0–38.7)	23.00 (12–39)	0.171
Serum hemoglobin (g/dL), mean ± SD	12.94 ± 2.16	13.08 ± 2.11	0.453

BUN: blood urea nitrogen; DBP: diastolic blood pressure; GFR: glomerular filtration rate; HF: heart failure; SBP: systolic blood pressure; BMI: body mass index; SD: standard deviation; bpm: beat per minute; Min: minimum; Max: maximum.

**Figure 1:**
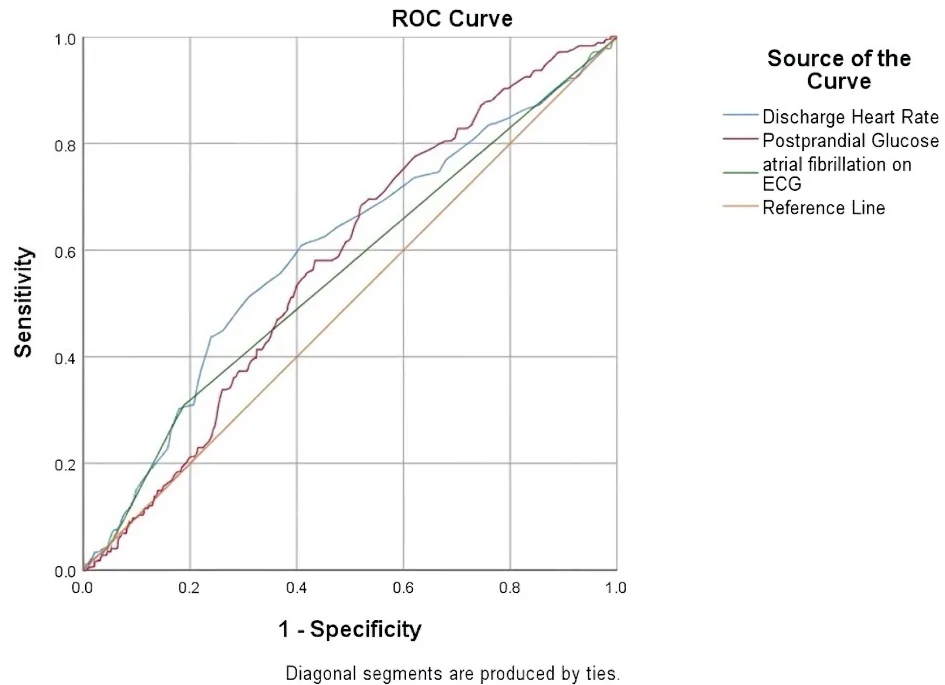
ROC curve of final predictor model of atrial fibrillation on ECG, HR on discharge and postprandial glucose. ROC: receiver operating characteristic; ECG: electrocardiogram; HR: heart rate.

**Figure 2:**
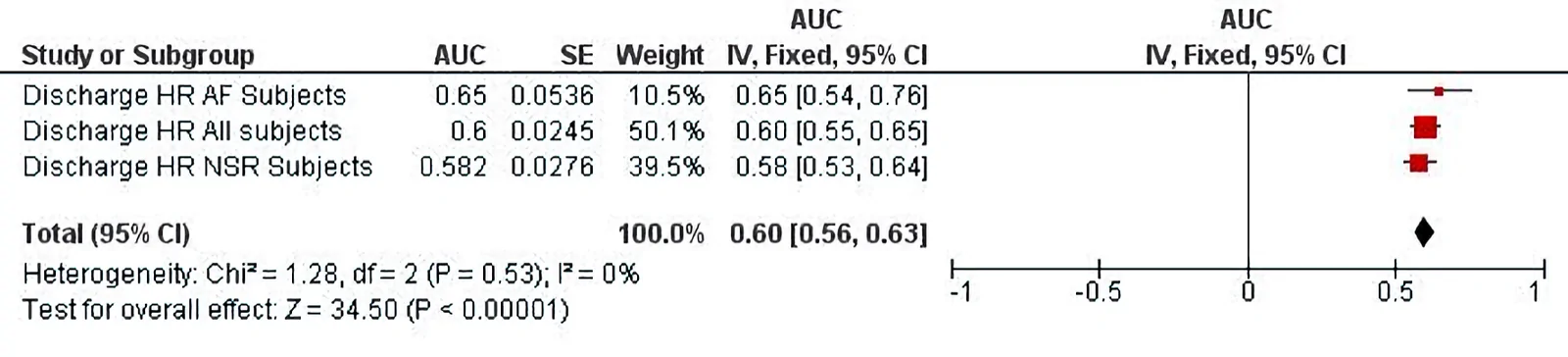
Forest plot comparing AUCs of discharge HR threshold of 76 bpm on all study subjects, subgroup analysis of HR threshold of 78 bpm on NSR study subjects, and subgroup analyses of HR threshold of 78 bpm on AF study subjects. AUC: area under the receiver operating characteristic (ROC) curve; HR: heart rate; bpm: beats per minute; NSR: normal sinus rhythm; AF: atrial fibrillation; CI: confidence interval; SE: standard error.

We performed logistic regression multivariate analysis using the backward Wald method. Our final model with a statistically significant result consisted of AF (odds ratio (OR) = 2.616; 95% CI, 1.604–4.267; P ≤ 0.001), and HR on discharge (OR = 1.022; 95% CI, 1.005–1.039; P = 0.01) as a predictor of readmission in 30 days, while postprandial glucose ≤ 140 mg/dL showed a protective effect against readmission in 30 days (OR = 0.528; 95% CI, 0.348–0.802; P = 0.003). We performed internal validation of the final multivariate model through bootstrapping (2,000 resamples, Mersenne Twister seed: 20000), which produced consistent results with all primary predictors retaining statistical significance and stable BCa 95% CI, confirming the statistical robustness of our analysis result. Table 4 shows the results of multivariate analysis. Receiver operating characteristic (ROC) analysis was performed on all study subjects pertaining to our final model of AF, HR on discharge, and postprandial glucose. The corresponding area under the receiver operating characteristic curve (AUROC) values were 0.557 (95% CI, 0.507–0.607; P = 0.022), 0.599 (95% CI, 0.549–0.648; P < 0.001), and 0.579 (95% CI, 0.533–0.624; P = 0.002), respectively (Fig. 1). We also perform a pooled analysis of all AUCs using a forest plot for AUCs concerning discharge HR thresholds across various study subject subroups (Fig. 2).

**Table 4:** Result of Multivariate Analysis on Factors Associated With Heart Failure Readmission in 30 Days

Variables	P	OR	95% CI	
			Minimum	Maximum
Atrial fibrillation on ECG	0.000	2.616	1.604	4.267
Heart rate on discharge	0.010	1.022	1.005	1.039
Postprandial glucose < 140 mg/dL	0.003	0.528	0.348	0.802

ECG: electrocardiogram; OR: odds ratio; CI: confidence interval.

ROC curve analyses were performed to evaluate the discriminative utility of discharge HR on readmission. The analyses showed an area under the ROC curve (AUC) of 0.600 (95% CI, 0.552–0.649); P ≤ 0.001), with sensitivity and specificity of 0.654 and 0.521, respectively, at a discharge HR threshold of 76 bpm.

Subgroup ROC curve analysis in normal sinus rhythm (NSR) patients on discharge HR vs readmission showed an AUC of 0.582 (95% CI, 0.528–0.637; P = 0.003), with sensitivity and specificity of 0.581 and 0.585, respectively, with a threshold of discharge HR of 78 bpm.

**Figure 3:**
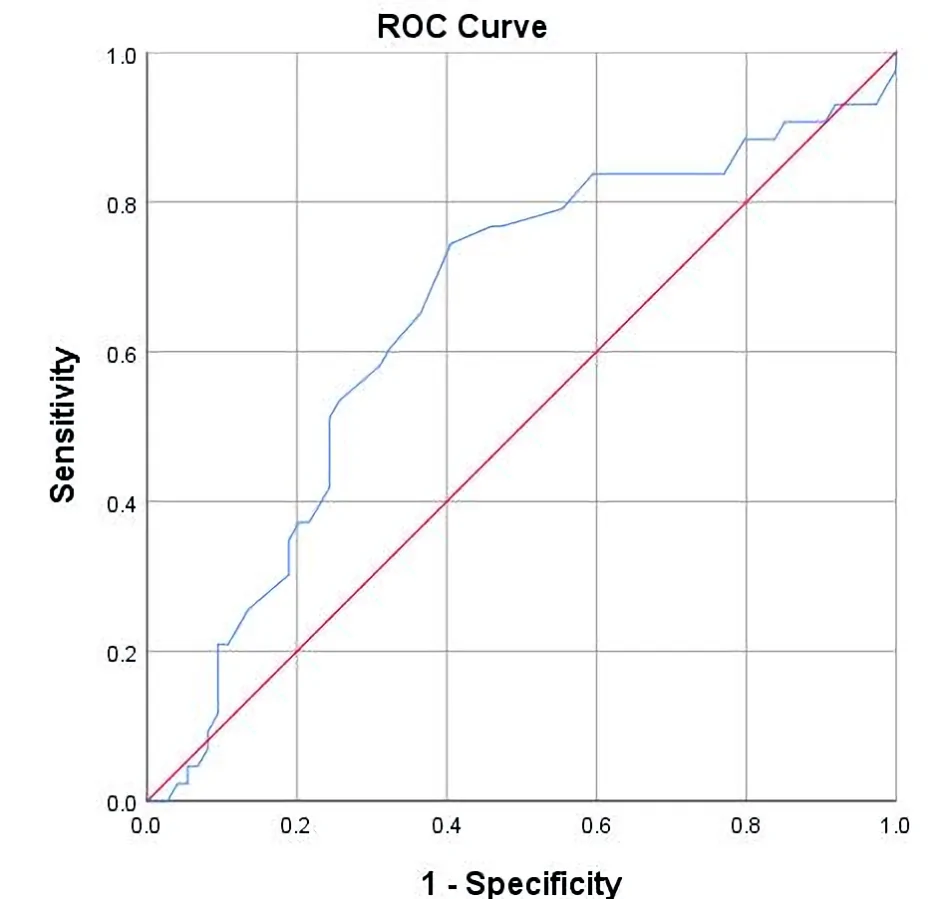
ROC curve of subgroup analysis between discharge HR and rehospitalization in diabetic ADHF patients with AF. ROC: receiver operating characteristic; HR: heart rate; ADHF: acute decompensated heart failure; AF: atrial fibrillation.

Subgroup ROC curve analysis in AF patients on discharge HR vs readmission showed an AUC of 0.650 (95% CI, 0.545–0.756; P = 0.007), with sensitivity and specificity of 0.744 and 0.595, respectively, with a threshold of discharge HR of 78 bpm. Figure 3 shows ROC analysis between discharge HR and rehospitalization in AF patients.

## Discussion

The main findings of this study are: (1) ADHF HFrEF patients with poor adherence, AF on admission, fasting blood glucose ≥ 100 mg/dL, postprandial glucose ≥ 140 mg/dL, higher median SBP, and higher BUN and Cr were associated with readmission; (2) Using multivariate analysis, ADHF HFrEF patients with AF, higher HR on discharge, and postprandial glucose ≥ 140 mg/dL were associated with readmission; (3) ROC analysis showed that in ADHF HFREF patients with AF, a target HR of 78 bpm showed modest discriminative ability against readmission, with an AUC of 0.650 (95% CI, 0.545–0.756; P = 0.007), with a sensitivity and specificity of 0.744 and 0.405; (4) ROC analysis showed that in ADHF HFrEF patients with AF and NSR, a target HR of 76 bpm showed modest discriminative ability against readmission, with an AUC of 0.600 (95% CI, 0.552–0.649; P ≤ 0.001), with sensitivity and specificity of 0.654 and 0.521, respectively.

In this study, we observed an incidence of 30-day readmission of 23.96% (n = 179). This result is similar to the previous study by Krumholz et al in the USA, which described readmission rates between 18% and 30% [8]. The median age of patients in this study is 58 years, which is similar to the study by Chamberlain et al [7] and Aranda et al [9], which showed the highest readmission rates in patients aged < 65 years old. Our result reflects the result of a previous study by Siswanto et al, which showed that the mean age of ADHF patients in Indonesia is 60 years old [4]. The median LOS in this study was 6 days in both groups. This is similar to a study by Samsky et al, which showed the mean LOS in readmitted ADHF patients to be 4.9 ± 3.7 days in the USA and 7.3 ± 5.6 days in Canada [10]. The incidence of infection during admission was quite high in our study (65.6%); however, during analysis, infection was not found to be affecting readmission rates or LOS. This result differs from a study by Alon et al, which showed higher readmission rates in ADHF patients with infection during admission [11]. Several factors could explain this disparity, including differences in treatment strategies, antimicrobial approaches, and clinical characteristics of the study populations.

CKD is present on 19.7% of all study subjects. However, CKD is not found to be associated with readmission rates on bivariate analysis, while Sherer et al [12] in their study found CKD to be associated with 30-day readmission rates in HF patients. This difference might be caused by the fact that a significant portion of our patients with CKD were new referrals to our hospital, and their CKD diagnoses were established only by history taking and anamnesis.

Lower usage of ACE-I/ARB/ARNI was seen in patients who were readmitted in 30 days in this study (88.3% vs 93.7%, P = 0.018). A similar result is also shown in a study by Sadiq et al [13], which reported that the absence of ACE-I/ARB at discharge was associated with a higher risk of readmission in 30 days (OR = 2.4, P = 0.03) [13]. Inhibition of the renin–angiotensin–aldosterone system (RAAS) plays a significant role in reducing readmission rates in HF patients. This principle also applies to HF patients with T2DM, in which sympathetic upregulation occurs in T2DM patients. Agents belonging to the ACE-I/ARB/ARNI class help suppress sympathetic activation in these patients [13].

One interesting finding in this study is that usage of beta-blockers, even when not associated with a reduction in readmission risk in 30 days, also resonated with other studies. A study by Bhatia et al [14] also showed no association between the administration of beta-blockers and a reduction in readmission risk. Although previous studies have shown beta-blockers to prevent maladaptive cardiac remodeling, which serves as the rationale for prescribing this agent, the protective effect of beta-blockers against rehospitalization might not be seen until up to 1 month after therapy, which might explain the lack of association between beta-blocker usage and reduction of readmission risk in 30 days since the previous admission [13]. This assumption is contradicted by a study by Loop et al [15], which showed a lower readmission risk in 30 days in patients with beta-blockers at discharge. This difference might be caused by a different study population between these two studies [15]. The European Society of Cardiology (ESC) in its guideline still recommends prescribing beta-blockers in HFrEF patients to reduce readmission and mortality secondary to worsening HF [16].

Most of the patients in our study received OHO or insulin (90.4%). Bivariate analysis showed no association between OHO/insulin usage and the reduction of readmission risk. This might be caused by suboptimal blood glucose control in the study subjects, which is reflected by fasting blood glucose levels and postprandial glucose levels in patients with and without readmission (113 (40–321 vs 104 (34–324), P = 0.015; and 158.5 (80–372) vs 142.5 (51–409), P = 0.002, respectively).

In this study, there were more patients with AF at admission in the readmission group compared to the non-readmission group (24% vs 13%, P = 0.001). AF at admission was also found to be associated with a higher readmission risk within 30 days from our multivariate analysis. This association was also shown in previous studies in patients with HF and AF [12, 17–19]. AF in HF cases might reflect a deterioration of ventricular function or an increase in neurohormonal activation. AF can also be the cause of new HF cases or worsening of HF cases. AF causes loss of atrial contractile function, which in turn will contribute to a decrease in cardiac output [20]. AF causes HF by a decrease in cardiac output and by tachycardia-induced cardiomyopathy (TIC) [20]. DM is also a risk factor for the incidence of AF. In diabetic patients, remodeling of the atrial structure by fibrosis and atrial dilatation serves as the main substrate for the incidence of T2DM-related AF. This remodeling is believed to be caused by a combination of metabolic factors such as oxidative stress, advanced glycation end-products (AGEs), and increased expression of growth factors [21]. Patients with T2DM are more at risk of AF [22]. Inadequate blood sugar control in T2DM patients also increases AF development in T2DM patients [22]. In the subpopulation of patients who experienced readmission in this study, out of 43 (24%) patients, 27 (62.8%) patients had fasting blood sugar > 100 mg/dL, and 21 (48.8%) patients had postprandial glucose levels > 140 mg/dL.

Higher resting HRs were seen in the readmission group compared to the non-readmission group (81 (50–114) vs 72 (51–105), P < 0.001). Resting HR was also found to be associated with higher readmission risk in our multivariate analysis (OR = 1.022; 95% CI, 1.005–1.039; P = 0.01). This finding is also similar to the result of two other studies by Kaneko et al [23] and Laskey et al [24]. In T2DM patients, an increase in resting HR is mediated by vagal inhibition and increased activation of the sympathetic axis. A resting HR > 80 bpm in patients with HF might contribute to further myocardial dysfunction. In the SHIFT (Systolic Heart Failure Treatment with the I_f_ Inhibitor Ivabradine Trial) study, an increase in resting HR was shown to be associated with increased cardiovascular mortality and rehospitalizations secondary to worsening of HF [25]. This myocardial dysfunction is also believed to be caused by the downregulation of beta receptors with suppression of signal transduction, disruption of intracellular calcium homeostasis, and excitation-contraction coupling. A persistent HR above 100 bpm might even cause TIC [26].

Diabetic patients with higher resting HRs are associated with a worse prognosis in terms of mortality and cardiovascular complications. Resting HR is positively correlated with hyperglycemia status in T2DM patients in terms of fasting blood glucose, postprandial glucose, and glycated hemoglobin (HbA1c). This association is mediated by autonomic system dysfunction secondary to high blood glucose levels [27].

We performed further subgroup analysis in ADHF patients with AF at admission ROC curve to investigate the relationship between discharge HR and readmission risk. ROC curve analysis showed an AUC of 0.650 (95% CI, 0.545–0.756; P = 0.007), with sensitivity and specificity of 0.744 and 0.405, respectively, with a threshold of discharge HR of 78 bpm. Currently, a target HR of 60–110 bpm is recommended for AF patients with acute or chronic HF [28]. Evidence regarding the impact of HR control in the prognosis of AF patients remains inconsistent, with studies showing equivocal results between lenient rate control vs strict rate control [29–31], and even no effect at all [30]. However, it is clear that in patients with HFrEF and AF, an HR above 100 bpm is associated with increased mortality in AF patients [32]. With these results, it might be beneficial to pursue a discharge HR below 78 bpm in diabetic patients with HFrEF and AF. On the other hand, there are still inconsistencies regarding impact of AF on in-hospital mortality of ADHF patients, with ASCEND HF study showing the association between AF and cumulative 30-day all-cause mortality and HF hospitalization [33], while results of HEARTs registry study showing equivocal results of mortality between AF and non-AF patients [34]. A study in Norwegian ADHF patients with AF also showed that AF was not associated with increased mortality rate in ADHF patients using multivariate analysis [35]. Nevertheless, analyses of three randomized controlled trials (RCTs) showed that a history of AF is associated with less loss of weight and a decrease in N-terminal pro–B-type natriuretic peptide (NT-proBNP) levels in ADHF patients, thus making decongestive efforts more difficult in ADHF patients with AF [36].

From our multivariate analysis, postprandial glucose levels < 140 mg/dL were shown to be associated with a lower risk of readmission in 30 days in patients with HFrEF and T2DM (OR = 0.528; 95% CI, 0.348–0.802; P = 0.003). Inadequate control of blood sugar in T2DM will cause endothelial dysfunction and cardiovascular comorbidities [20]. Postprandial glucose levels, not fasting glucose levels, have also been shown to be associated with cardiovascular adverse events in both men and women [37]. In diabetic patients, systemic, myocardial, and cellular mechanism plays a role in causing structural heart disease and HF [38]. Apart from tendencies to cause ischemia/myocardial infarction, diabetes also causes myocardial dysfunction in the absence of coronary heart disease, a condition named diabetic cardiomyopathy [39]. Imaging studies have shown left ventricular hypertrophy to be a characteristic finding in patients with T2DM. Left ventricular hypertrophy causes diastolic dysfunction, which is an early manifestation of diabetic cardiomyopathy. This finding is present in 40–75% of diabetic patients [39].

Our postprandial blood glucose cutoff was set at 140 mg/dL, while our optimal discharge HR thresholds (68–76 bpm) were mathematically derived via ROC curve analysis to maximize diagnostic sensitivity and specificity. Our cutoff for postprandial blood glucose was based on thresholds by the American Diabetes Association guideline; Our discharge HR threshold was supported by major guidelines from ESC/American Heart Association (AHA) and the landmark clinical trial of the RACE II trial, which explicitly established < 80 bpm as the strict physiological boundary for resting HR control [29, 40].

Compared to the classical clinical course of HF, the current clinical trajectory of HF patients is heavily influenced by the post pandemic burden of coronavirus disease 2019 (COVID-19) and its long-term sequelae of “long COVID”. Recent literature has discussed the emerging evidence that severe acute respiratory syndrome coronavirus 2 (SARS-CoV-2) can act as a chronic accelerator, worsening cardiovascular outcomes in patients with cardiovascular comorbidities. A recent systematic review by Parizad et al (2026) [41] showed that patients with pre-existing HF showed an increased rate of readmission related to worsening HF following COVID-19 infection. The authors of the review postulated that the sustained post viral vulnerability is associated with low-grade myocardial inflammation, systemic inflammation, and endothelial injury. Furthermore, when these pathological mechanisms occur in patients with concomitant T2DM, the risk is further increased, as shown in a study by Zhao et al (2025), who described a bidirectional interplay between SARS-CoV-2 infection and diabetes [42]. The authors of these studies postulated that the virus induced glycemic destabilization, alteration in lipid, and coagulation metabolism, which affect cardiovascular complications [29, 41].

Chronic hyperglycemia in T2DM patients causes the generation of AGEs, which in turn will cause cross-linking in collagen molecules and ultimately cause intramyocardial fibrosis. This fibrosis will reduce myocardial elasticity and disrupt cardiac relaxation. Diabetes also causes maladaptive calcium homeostasis and stress on the endoplasmic reticulum, which also contributes to myocardial fibrosis. Chronic hyperglycemia also contributes to activation of the RAAS system, leading to increased secretion of angiotensin II and aldosterone, which also contributes to cardiac hypertrophy and myocardial fibrosis [39].

### Study limitations

Our study has several limitations. First, due to the nature and constraints of retrospective single-center design at a national referral center, the findings of this study might have limited external generalizability to primary or community care settings. Furthermore, while our final sample of 747 clinical encounters provided sufficient statistical power for multivariate analysis, this number still represents a modest cohort compared to larger-scale multicenter registries. With the retrospective nature of the analysis, we were also unable to account for unmeasured socioeconomic factors, which may independently influence the risk of 30-day readmission regardless of the patient’s clinical or metabolic status.

Second, regarding discharge medication and the implementation of guideline-directed medical therapy (GDMT), a high number of our clinical encounters received GDMT (92.4% received an ACE-I/ARB/ARNI, 82.3% received a beta-blocker, and 68.3% received an MRA). Due to the retrospective nature of the date, we could not verify what percentage of these patients achieved true maximum GDMT doses. Sodium-glucose cotransporter 2 inhibitors (SGLT2i) were not evaluated as a class of the four pillars of the HFrEF therapy because our study was conducted from January 2016 to February 2021, in which time the SGLT2i were not readily available in Indonesian HF centers and were not included in local HF guideline. The lack of standardized SGLT2i therapy strictly reflects the historical timeline of regional guidelines and availability rather than clinical omission.

Third, this study lacked longitudinal post-discharge tracking to correlate 30-day readmission outcomes directly with long-term medication compliance. The high rate of missing data in the medication adherence variable necessitated the exclusion of this variable from our analysis to ensure data integrity. There is still a possibility that unmeasured confounding of post-discharge non-compliance may have independently caused early rehospitalization.

Fourth, not all subjects in our study received a uniform or comprehensive diagnostic workup for CKD, such as standardized baseline laboratory assays and renal ultrasonography at every presentation. This may have introduced a risk of misclassification bias regarding CKD in our study subjects, which might explain why CKD status was not observed to be significantly associated with readmission risk. Finally, a statistical limitation of this study is the inclusion of multiple admissions from the same individual patient, which could potentially affect the assumption of independence required for standard logistic regression modelling. However, our internal validation of our primary predictors using bootstrapping generated highly stable CIs, which confirms excellent consistency of the identified clinical and metabolic risk factors across successive clinical encounters.

### Conclusions

This study showed that clinical factors such as AF and increased HR at discharge were associated with rehospitalization risk in 30 days in patients with HFrEF and T2DM, while a metabolic factor of postprandial blood sugar ≤ 140 mg/dL was observed to have protective effects against rehospitalization in 30 days in patients with HFrEF and T2DM. ROC analysis showed that reducing discharge HR to below 78 bpm might be beneficial in diabetic patients with HFrEF and AF.

### Learning points

ADHF patients with HFrEF face high 30-day readmission rates, particularly when complicated by T2DM, which increases cardiovascular complexity and mortality risk. Existing risk scores often fail to reflect the clinical profiles of non-Western populations.

This study identifies AF, high discharge HR, and postprandial glucose < 140 mg/dL as key readmission predictors in an Indonesian cohort. Future implications suggest that targeting a discharge HR < 78 bpm and strict postprandial glycemic control could significantly reduce rehospitalization.

## Data Availability

The data supporting the findings of this study are available from the corresponding author upon reasonable request.

## Abbreviations

ACE-I: angiotensin-converting enzyme inhibitor
AGEs: advanced glycation end-products
AF: atrial fibrillation
ARB: angiotensin receptor blocker
ARNI: angiotensin receptor neprilysin inhibitor
BUN: blood urea nitrogen
CKD: chronic kidney disease
DBP: diastolic blood pressure
ECG: electrocardiogram
EF: ejection fraction
GFR: glomerular filtration rate
HFrEF: heart failure with reduced ejection fraction
LOS: length of stay
NSR: normal sinus rhythm
SBP: systolic blood pressure
SR: sinus rhythm
VT: ventricular tachycardia
T2DM: type 2 diabetes mellitus
TIC: tachycardia-induced cardiomyopathy
